# New Role of *P. brasiliensis* α-Glucan: Differentiation of Non-conventional Dendritic Cells

**DOI:** 10.3389/fmicb.2019.02445

**Published:** 2019-10-30

**Authors:** Ana Camila Oliveira Souza, Cecília Favali, Naiara Caroline Soares, Natalia Machado Tavares, Márcio Sousa Jerônimo, Paulo Henrique Veloso Junior, Clara Luna Marina, Claire Santos, Cláudia Brodskyn, Anamelia Lorenzetti Bocca

**Affiliations:** ^1^Departamento de Biologia Celular, Universidade de Brasília, Brasília, Brazil; ^2^Centro de Pesquisas Gonçalo Moniz, Fundação Oswaldo Cruz, Salvador, Brazil

**Keywords:** Paracoccidiodomycosis, *Paracoccidioides brasiliensis*, dendritic cell differentiation, glucan, cell wall fractions

## Abstract

The cell wall has a critical role in the host immune response to fungal pathogens. In this study, we investigated the influence of two cell wall fractions of the dimorphic fungi *Paracoccidioides brasiliensis* (Pb) in the *in vitro* generation of monocyte-derived dendritic cells (MoDCs). Monocytes were purified from the peripheral blood of healthy donors and cultivated for 7 days in medium supplemented with IL-4 and GM-CSF in the presence of Pb cell wall fractions: the alkali-insoluble F1, constituted by β-1,3-glucans, chitin and proteins, and the alkali-soluble F2, mainly constituted by α-glucan. MoDCs phenotypes were evaluated regarding cell surface expression of CD1a, DC-SIGN, HLA-DR, CD80, and CD83 and production of cytokines. The α-glucan-rich cell wall fraction downregulated the differentiation of CD1a^+^ MoDCs, a dendritic cell subset that stimulate Th1 responses. The presence of both cell fractions inhibited DC-SIGN and HLA-DR expression, while the expression of maturation markers was differentially induced in CD1a^–^ MoDCs. Differentiation upon F1 and F2 stimulation induced mixed profile of inflammatory cytokines. Altogether, these data demonstrate that Pb cell wall fractions differentially induce a dysregulation in DCs differentiation. Moreover, our results suggest that cell wall α-glucan promote the differentiation of CD1a^–^ DCs, potentially favoring Th2 polarization and contributing to pathogen persistence.

## Introduction

The cell wall is a complex and dynamic structure that plays essential roles in fungal cell biology. Cell shape, polarized growth, sensing of, response to or protection against surrounding conditions and interaction with the extracellular environment are some of the processes fully dependent on a healthy fungal cell wall. This organelle is a polysaccharide-rich shield that surrounds the outer face of the phospholipidic cell membrane, and its molecular composition and structure varies according to fungal species, morphotype and life cycle. In most fungi, the cell wall is organized as a structural scaffold formed by cross-linked β-1,3-glucan, β-1,6-glucan and chitin, embedded in a more heterogeneous amorphous matrix, often constituted by α-1,3-glucan and mannan. Besides, proteins are often covalently linked to those carbohydrates. Importantly, cell wall components are mostly absent in animals and plants. For this reason, it is an important target for the development of antifungal therapy and its contribution to host immune responses has been vastly investigated. The composition and structure of the fungal cell wall have been described in several other review papers (Bowman and Free, 2006; Latgé, 2010; Free, 2013; Gow et al., 2017).

In the fungal pathogen *Paracoccidioides brasiliensis* (Pb), the development and maintenance of the Pb cell wall is somewhat peculiar due to its thermo-dimorphic nature. Similarly to the other species in the *Paracoccidioides* genus, *P. brasiliensis* grow as a mold in the environment (18–25^°^C) and at higher temperatures (35–37^°^C), it switches to a multi-budding yeast cell morphology (Almeida and Lopes, 2001; Shikanai-Yasuda et al., 2006, 2017; Calich et al., 2008; Ferreira, 2009; Fernandes et al., 2015; Turissini et al., 2017). As such, *P. brasiliensis* environmental fungal propagules can be inhaled and transformed into pathogenic yeasts once inside the host tissues. Not surprisingly, differences in the structure and composition of the cell wall during mycelial or yeast growth have been previously reported. The environmental mycelia cell wall presents higher amounts of proteins and β-glucan, while the pathogenic yeast cell wall is thicker and bears higher amounts of chitin (inner layer) and α-1, 3-glucan (95% of total glucan, outer layer) (Kanetsuna et al., 1969; Moreno et al., 1969; Kanetsuna and Carbonell, 1970; San-Blas and San-Blas, 1977; Nóbrega et al., 2010). The protrude presence of peripheral α-glucan in Pb pathogenic yeast is believed to play a role in pathogenicity (Kanetsuna and Carbonell, 1970; San-Blas and San-Blas, 1977; San-Blas et al., 1977), similar to other fungal pathogens, such as *Histoplasma capsulatum* and *Blastomyces dermatitidis* (Klimpel and Goldman, 1988; Hogan and Klein, 1994).

*Paracoccidioides brasiliensis* is one of the etiologic agents of Paracoccidiodomycosis (PCM), a severe, non-opportunistic granulomatous mycosis endemic in Latin America that, when not quickly diagnosed and adequately treated, can result in a disseminated and life-threatening disease (Bocca et al., 2013). It has been extensively demonstrated that a sustained secretion of Th1 cytokines plays a dominant role in the mechanism of resistance to Pb infection, while an intense humoral response is associated with increased disease dissemination and severity (Almeida and Lopes, 2001; Silvana dos Santos et al., 2011; Camacho and Niño-Vega, 2017). As key players in the development of adaptive immune responses, dendritic cells (DCs) recognize Pb antigens and migrate to lymph nodes, where they activate T helper cells (Silvana dos Santos et al., 2011; Tavares et al., 2012; Pina et al., 2013). However, it has been reported that Pb infection can cause impairment in DCs maturation, leading to a non-adequate cell-mediated response that contributes to the host susceptibility to this pathogen (Ferreira et al., 2004, 2007; Ferreira and Almeida, 2006; Fernandes et al., 2015). Therefore, in this study we analyzed the contribution of two Pb cell wall fractions in the *in vitro* differentiation and maturation of DCs generated from human monocyte cells. Our results demonstrate that both alkali-insoluble β-glucan rich (F1) and alkali-soluble α-glucan rich (F2) cell wall fractions differentially alter *in vitro* differentiation of human monocyte-derived DCs regarding the expression of HLA-DR, DC-SIGN, CD83, and CD80 molecules, and their ability to secrete inflammatory cytokines. Importantly, our findings suggest that Pb cell wall α-glucan stimulates the differentiation of CD1a^–^ DCs, what can affect polarization of Th1 immune response and play a role in pathogenesis.

## Materials and Methods

### Fungal Strains and Culture

The highly virulent (Pb18) and avirulent (Pb265) strains of *P. brasiliensis* were obtained from the fungal collection of the Applied Immunology Laboratory at the Biology Institute of the University of Brasilia. The fungus was cultured in liquid YPD medium (w/v: 2% peptone, 1% yeast extract, 2% glucose) at 36°C in a rotary shaker (220 rpm). After 5 days of growth, the fungal cells were harvested by centrifugation, washed with PBS and the cell pellet was further used for experiments.

### Flow Cytometry Analysis of β-Glucan Exposure

Characterization of β-glucan exposure in Pb18 and Pb265 isolates was performed by flow cytometry. First, 2 × 10^6^ yeast were harvested, resuspended in PBS 1% BSA (Sigma-Aldrich, St. Louis, MO, United States) supplemented with 2% FBS (Gibco, United States) and incubated with polyclonal anti-β-glucan (1:100) for 1 h at 4^°^C. The polyclonal anti-β-glucan was obtained from mice serum after three consecutive immunizations with depleted zymosan (#tlrl-zyd, InvivoGen, CA, United States) emulsified with Freund’s incomplete adjuvant (Sigma-Aldrich, MO, United States) and purified using protein-A column, as described previously (Leenaars and Hendriksen, 2005). Next, the yeasts were washed three times and incubated for 1 h with a polyclonal goat anti-mouse IgG conjugated with fluorescein isothiocyanate (FITC) (1:400 eBioscience), used according the manufacturer’s recommendations. *Cryptococcus neoformans* encapsulated strain (H99) was used as a negative control while acapsular strain (Cap65) was used as a positive control (data not shown). Isotypic FITC-conjugated antibody was also used as a control. Cells were acquired in the FACSVerse flow cytometer (BD Biosciences), and the data analyzed in the FlowJo software (version X).

### Chemical Fractionation of Pb Cell Wall

Alkali-treatment was utilized to separate the cell wall components of Pb into two fractions: an alkali-insoluble fraction constituted by chitin, β-glucans and amino acids (F1) and an alkali-soluble fraction that is precipitated with acetic acid and constituted mainly by α-glucan (F2). The protocol for Pb cell wall fractionation was followed according to previous reports (Kanetsuna and Carbonell, 1970; San-Blas and San-Blas, 1977; Nóbrega et al., 2010; Puccia et al., 2011). Briefly, after harvesting, yeast cells were inactivated with 5% formaldehyde for 24 h, filtered onto tissue paper, washed three times with distilled water and dried at room temperature. Then, the material was frozen with liquid nitrogen in order to rupture fungal cells, diluted in distilled water and washed five times by centrifugation at 2000 *g*. The raw cell wall material was then subjected to lipid extraction with chloroform:methanol (2:1 v/v). The lipid-free cell wall material was precipitated with alkali solution (1N NaOH) and gently stirred at room temperature for 1 h. After centrifugation at 5000 *g* for 10 min, the supernatant (alkali-soluble) was collected and the procedure was repeated four times more with the remaining precipitate (alkali-insoluble). The alkali-insoluble precipitate was washed with water until it reached pH 7.0, and then sequentially washed with ethanol, acetone and diethyl ether (v/v/v). The resulting white powder consisted in the alkali-insoluble F1 fraction, constituted mainly by β-glucans, chitin and proteins (Kanetsuna and Carbonell, 1970; Nóbrega et al., 2010; Puccia et al., 2011). The amount of F1 (300 μg/ml) used in these experiments was based on our previous results from migration kinetics experiments (Nóbrega et al., 2010).

To obtain F2, the combined supernatants obtained after alkali extraction of F1 were neutralized by the addition of chloridric acid (1 M HCl). The precipitate was washed three times with distilled water and further diluted in 0.1 M HCl. After successive washes, the insoluble precipitate was collected, resuspended in water and dialyzed against distilled water, resulting in an alkali-soluble F2 fraction, acid precipitable and mostly constituted by α-1,3-glucans (Kanetsuna and Carbonell, 1970; Nóbrega et al., 2010; Puccia et al., 2011). The material was lyophilized and re-suspended to a final concentration of 1 mg/mL. The amount of F2 used in these experiments (450 μg/ml) was defined using our previous results from migration kinetics experiments (data not shown).

### Nuclear Magnetic Resonance (NMR) Spectroscopy

The cell wall fractions F1 (1.2 mg) and F2 (1.1 mg) were deuterium-exchanged by lyophilization in D_2_O. The dried samples were re-dissolved in 0.5 mL D_2_O (99.96% D, Cambridge Isotope Laboratories) and transferred to a 5-mm OD NMR tube. 1-D Proton spectra were acquired on a Varian Inova-500 MHz spectrometer at 25°C. These analyses were performed by the Complex Carbohydrate Research Center (GA, United States).

### *In vitro* Generation of Human Monocyte-Derived Dendritic Cells

Immature monocyte-derived DCs were generated from peripheral blood monocytes (PBMC) obtained from 12 healthy donors (Blood Center of Salvador, HEMOBA, Brumado, Brazil authorization number 100/2006). Briefly, PBMC were obtained from heparinized venous blood by passage over a Ficoll Hypaque gradient (Sigma-Aldrich, St. Louis, MO, United States). PBMC were washed three times, and the CD14^+^ cell population was enriched by positive selection using magnetic cell sorting (Mini Macs, Miltenyi Biotec, Auburn, CA, United States). Monocytes were resuspended at a concentration of 5 × 10^5^ cells/ml in RPMI-1640 medium (Gibco, Grand Island, NY, United States) supplemented with 2 mM L-glutamine, penicillin (100 U/ml), streptomycin (100 g/ml, Gibco, Grand Island, NY, United States), and 10% heat-inactivated Fetal Bovine Serum (Cripion Biotechnology, Andradina, SP, Brazil), plus IL-4 (100 UI/ml) and GM-CSF (50 ng/ml; PeproTech, Rocky Hill, NJ, United States). Cells were cultured in 24-well tissue plates (Costar, Corning, NY, United States) and incubated at 37°C 5% CO_2_ for 7 days. Cell wall fractions F1 (300 μg/ml) or F2 (450 μg/ml) were added on the first day of culture to study the effect of these components on dendritic cell differentiation. At days 3 and 6, fresh medium was replaced with GM-CSF and IL-4 without further addition of antigens. After 7 days, in order to characterize the DCs population, cells were stained and fluorescence was analyzed by FACS (FACSort, Becton Dickinson, San Jose, CA, United States). Supernatants were harvested and maintained at −20^°^C for cytokine measurement.

### Immunophenotyping of MoDCs

After culture, immature DCs were harvested for flow cytometry analyses. Briefly, 10^6^ cells were incubated with PBS/1% BSA (Sigma-Aldrich, St. Louis, MO, United States)/0.1% sodium azide (Nuclear) and incubated with 20% FCS to block FcR (Mazurek et al., 2012). Cells were stained with FITC-conjugated anti-CD1a (clone HI149, Ebiosciences, San Diego, CA, United States), PE-CD80 (clone 2D10.4, Ebiosciences, San Diego, CA, United States), PECy5-CD83 (clone HB15e, BD Biosciences, San Jose, CA, United States), PECy-5-HLA-DR (clone LN3, Ebiosciences, San Diego, CA, United States) and PE-anti-DC-specific ICAM-grabbing non-integrin (DC-SIGN, clone DCN46, BD Biosciences, San Jose, CA, United States). All analyses included the appropriate isotype controls. Cells were acquired on FACSort (BD Biosciences, San Jose, CA, United States), and analyzed with FlowJo (10.0.6 Treestar, Ashland, OR, United States). Cells were gated into CD1a^+^ and CD1a^–^, and analyzed separately concerning the expression of DC-SIGN, CD80, CD83, and HLA-DR.

### Cytokine Bead Array (CBA)

Cytokines on supernatants obtained in the last 24 h of culture were measured by flow cytometry employing BD Cytometric Bead Array (CBA, BD Biosciences, San Jose, CA, United States) Human Inflammatory Cytokine Kit for IL-8, IL-1 beta, IL-6, IL-10, TNFα, and IL-12 p70 detection according to the manufacturer’s protocol.

### Statistical Analysis

Data were analyzed using GraphPad Prism (5.0, San Diego, CA, United States). Results were expressed as the mean ± SEM, and analyzed by *t*-test with Wilcoxon signed rank test; ^∗^*p* < 0.05, ^∗∗^*p* < 0.01, and ^∗∗∗^*p* < 0.001 in comparison to control cells. #*p* < 0.05, ##*p* < 0.01, and ###*p* < 0.001 between samples treated with the cell wall fractions.

## Results

### *Paracoccidioides brasiliensis* Strains Show Differences on β-Glucan Exposure

**FIGURE 1:**
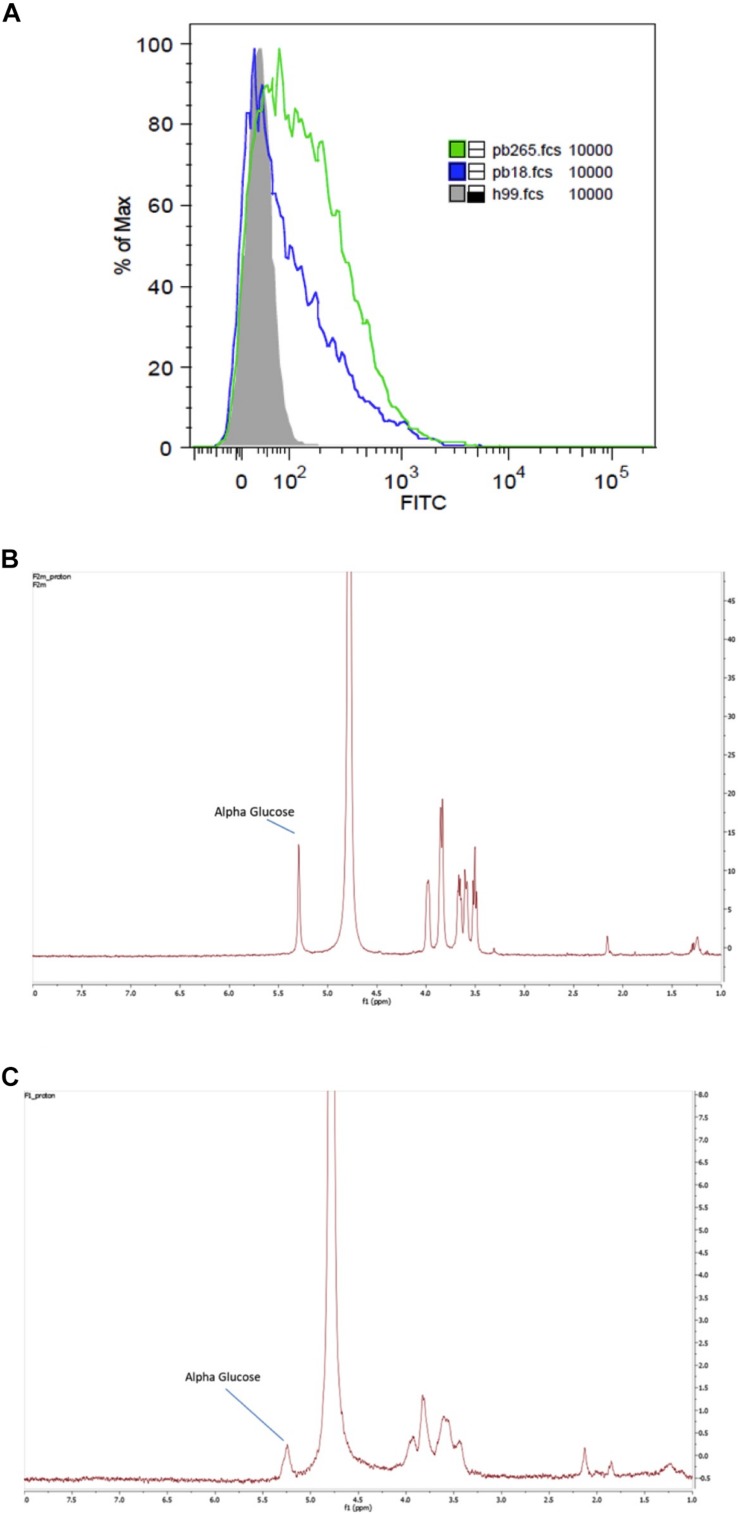
Cell wall α-glucan correlates to decrease in β-glucan exposure in *Paracoccidioides brasiliensis*. Exposure of β-glucan in the cell wall of *P. brasiliensis* virulent (Pb18) and avirulent (Pb265) isolates and *Cryptococcus neoformans* (H99) **(A)**. NMR spectroscopy of F2 **(B)** and F1 **(C)** cell wall fractions.

*Paracoccidioides brasiliensis* yeast cell wall is organized in an inner layer, mostly composed by β-glucan, chitin and proteins, and an outermost layer, which is mainly composed by α-glucan (Moreno et al., 1969; Carbonell et al., 1970; Kanetsuna and Carbonell, 1970; Puccia et al., 2011). As previously described for *H. capsulatum* (Klimpel and Goldman, 1988) and *B. dermatitis* (Hogan and Klein, 1994), the presence of α-glucan in the cell wall often correlates with virulence, possibly because it hinders immune recognition of β-glucan (Rappleye et al., 2007). In order to confirm the correlation of β-glucan exposure in the cell wall with Pb virulence, we sought to analyze two Pb isolates with well-known disparate virulence profiles: Pb265 (non-virulent) and Pb18 (virulent). The β-glucan staining revealed that the non-virulent Pb265 isolate presented higher levels of β-glucan exposure in the cell surface than the virulent strain Pb18 (Figure 1). This result corroborates previous studies that demonstrated that Pb265 stimulated higher cell recruitment to mice peritoneal cavity, as well as that the inoculation of F1 fraction of Pb265 induced a higher subcutaneous nodular formation when compared to Pb18 (Silva et al., 1994).

Next, we analyzed the presence of α-glucan in the alkali-insoluble (F1) and alkali-soluble (F2) fractions of Pb18 yeast cell wall by NMR. While the fraction F2 completely dissolved in the D_2_O, sample F1 was mostly insoluble. Therefore, F1 D_2_O suspension was centrifuged and only the supernatant was evaluated by NMR. In each NMR spectrum presented at Figures 1B,C, there are peaks between 5.36 and 3.81 ppm, which represent the α-glucose anomeric protons present in the α-glucan (H-1: 5.36; H-2: 3.64; H-3: 3.95; H-4: 3.64; H-5: 3.84; H-6: 3.81). Thus, F2 sample (Figure 1B) shows well-resolved α-glucose peak, and since this fraction was completely dissolved in D_2_O, the presence of β-glucose polymers was ruled out (Figure 1B). The F1 fraction showed a less-resolved α-glucose peak with much lower intensity (Figure 1C). Considering these results, we confirm previous reports that demonstrated that F2 fraction is mainly composed by α-glucan.

### *Paracoccidioides brasiliensis* α-Glucan-Rich Cell Wall Fraction Alter Differentiation of CD1a^+^ MoDCs

**FIGURE 2:**
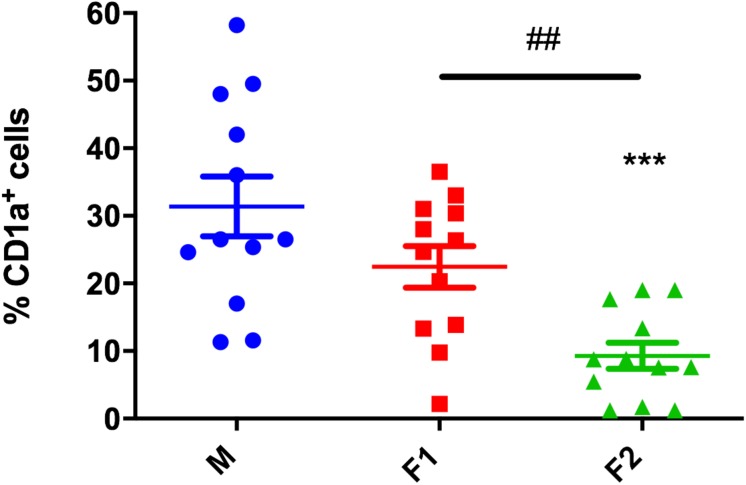
*Paracoccidioides brasiliensis* α-glucan-rich cell wall fraction modulates differentiation of CD1a^+^ dendritic cells. Cell frequency of CD1a+ cells after differentiation upon F1 or F2 stimulation. Non-stimulated cells were used as a control (M). Each data point represents one donor. Wilcoxon matched pairs *T* test. ^∗∗∗^*p* < 0.01 in comparison to control group. ^##^*p* < 0.01 between groups treated with cell wall fractions.

In order to elucidate if *P. brasiliensis* cell wall fractions could alter human DCs generation, monocytes were differentiated in the presence of F1 or F2 cell wall fractions. Monocytes not stimulated with cell wall fractions were used as a control (M). After 7 days of culture, we evaluated the expression of differentiation and maturation markers of DCs. First, we evaluated the differentiation of MoDCs regarding the expression of CD1a. This marker discern two DC subsets: CD1a^+^ DCs favor Th1 polarization, whereas CD1a^–^ DCs induce Th2 immunity (Chang et al., 2000; Cernadas et al., 2009). The F2 fraction downregulated the differentiation of CD1a^+^ MoDCs (9.2% ± 6.6%) in comparison with the non-treated samples (31.3 ± 15.3%) (Figure 2). On the other hand, the F1 fraction presence did not influence the levels of CD1a^+^ cells (Figure 2).

### *Paracoccidioides brasiliensis* Cell Wall Fractions Alter MoDCs CD1a Subset Maturation Phenotype

**FIGURE 3:**
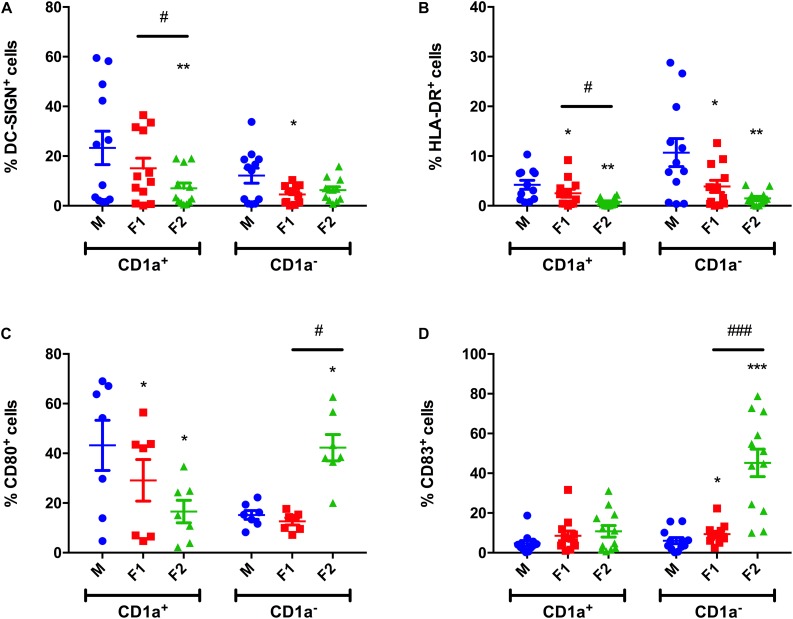
*Paracoccidioides brasiliensis* cell wall fractions modulate CD1a subsets phenotype. Frequency of DC-SIGN^+^ **(A)**, HLA-DR^+^ **(B)**, CD80^+^ **(C)**, and CD83^+^ **(D)** cell populations after differentiation upon F1 or F2 stimulation. Non-stimulated cells were used as a control (M). Each data point represents one donor. Wilcoxon matched pairs *T* test. ^∗^*p* < 0.05, ^∗∗^*p* < 0.01, and ^∗∗∗^*p* < 0.001 in comparison to control group. ^#^*p* < 0.05 and ^###^*p* < 0.001 between groups treated with cell wall fractions.

Since we observed that F2 downregulated the development of CD1a^+^ cells, we decided to evaluate both CD1a^+^ and CD1a^–^ subsets separately regarding the expression of specific surface molecules. F2 inhibited the DC-SIGN expression in CD1a^+^ cells, when compared with the control group (Figure 3A), whereas F1 decreased DC-SIGN expression in CD1a^+^ MoDCs (Figure 3A). Besides, both fractions induced significant decrease in HLA-DR expression in both DCs populations (Figure 3B). Inhibition of DC-SIGN and HLA-DR indicate negative modulation of antigen presentation (Chudnovskiy et al., 2019). CD80 expression in CD1a^+^ cells was inhibited by both cell wall fractions (Figure 3C). However, in CD1a^–^ cells, increased expression of CD80 (by F2 fraction, Figure 3C) and CD83 (both cell wall fraction, Figure 3D) was observed. Altogether, these data demonstrate that Pb cell wall fractions alter CD1a subsets phenotype, downregulating maturation of CD1a^+^ population. On the other hand, as cell wall fractions downregulate antigen presentation, they enhance expression of co-stimulatory molecules in the CD1a^–^ subset.

### *Paracoccidioides brasiliensis* Cell Wall Fractions Alter Human MoDCs Inflammatory Cytokines Secretion

**FIGURE 4:**
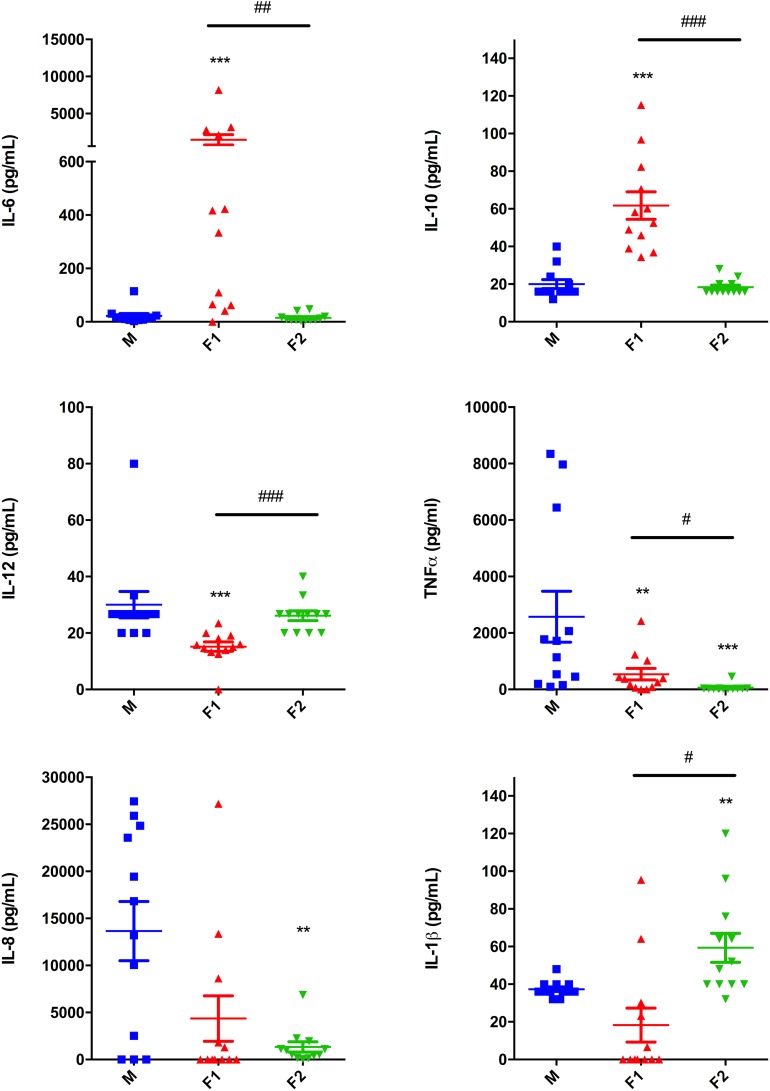
*Paracoccidioides brasiliensis* cell wall fractions modulate cytokine production by dendritic cells. IL-6, IL-10, IL-12, TNF, IL-8, and IL-1β levels were measured in the cell culture supernatants after differentiation upon F1 or F2 stimulation. Non-stimulated cells were used as a control (M). Each data point represents one donor. Wilcoxon matched pairs *T* test. ^∗∗^*p* < 0.01 and ^∗∗∗^*p* < 0.001 in comparison to control group. ^#^*p* < 0.05, ^##^*p* < 0.01, and ^###^*p* < 0.001 between groups treated with cell wall fractions.

At last, we analyzed the profile of inflammatory cytokines produced in the last 24 h of DCs differentiation under stimulation with Pb cell wall fractions. We observed that differentiation upon F1 stimulation induced an increase in IL-6 and IL-10 levels, while decreasing production of IL-12 and TNFα (Figure 4). Meanwhile, F2 stimulation induced a decrease in TNFα and IL-8, but significantly induced IL-1β expression. These data indicate that Pb cell wall fractions differentially modulate the ability of inflammatory cytokine production by MoDCs.

## Discussion

Dendritic cells are the most specialized antigen-presenting cells and have a crucial role in directing appropriate immune responses to favor PCM resolution. The protection against PCM is related to a prevalent cell-mediated immune response, with production of Th1 cytokines and activation of macrophages and lymphocytes that induce fungal killing and isolation in well-defined granulomas (Borges-Walmsley et al., 2002; Ferreira, 2009; Bocca et al., 2013). On the other hand, polarization toward a Th2 immune response enhances the disease (Bocca et al., 2013; Camacho and Niño-Vega, 2017; Shikanai-Yasuda et al., 2017). Given the importance of DCs in directing T-cell responses and immune response polarization, we analyzed the impact of Pb cell wall fractions in the differentiation and maturation of human monocyte-derived DCs.

Early studies show that in the *P. brasiliensis* pathogenic yeast the majority of cell wall glucans are α-linked, while only 4% are β-linked (Kanetsuna et al., 1969, 1972). β-glucans form a fibrillary scaffold that stabilizes the cell wall and are likely found closer to the cell membrane (Puccia et al., 2011; Latgé, 2017). On the other hand, α-glucan is embedded in the cell wall as an amorphous substance, forming a prominent outlayer (Puccia et al., 2011; Latgé, 2017). Reduction of cell wall α-glucan severely attenuates virulence in murine models of *H. capsulatum* (Klimpel and Goldman, 1988), *B. dermatitis* (Hogan and Klein, 1994), and *P. brasiliensis* infection (San-Blas et al., 1977). Likewise, abundance of α-glucan in *P. brasiliensis* pathogenic yeasts and its absence in non-pathogenic mycelial forms also corroborates the correlation of this polysaccharide to fungal virulence (San-Blas and San-Blas, 1977). Our results demonstrated that a non-virulent isolate of *P. brasiliensis* presented higher exposure of β-glucan in comparison to a virulent-isolate, possibly because α-glucan masks the inner layer of the cell wall, preventing immune recognition of β-glucan (Sukhithasri et al., 2013). Similarly, the presence of α-glucan on the *H. capsulatum* cell wall blocked the host recognition of β-glucan by dectin-1, reducing TNFα production and avoiding proper activation of the immune system (Rappleye et al., 2007).

Our data demonstrates, however, that besides hindering exposure of β-glucans, α-glucans can also negatively modulate differentiation of CD1a^+^ DCs. We analyzed CD1a expression after DC differentiation in the presence of cell wall fractions. CD1a is a well described DCs subset marker involved in the presentation of lipid or lipid-based molecules (Cernadas et al., 2009). CD1a^+^ DCs produce high levels of IL-12 and can polarize Th1 cells, whereas CD1a^–^ DCs lack IL-12 production and secrete high levels of IL-10, favoring development of Th2 immunity (Chang et al., 2000; Cernadas et al., 2009). DCs differentiation upon F2 led to a significant decrease in CD1a^+^ cells, suggesting that α-glucan may play a role in Th2 polarization during PCM. The same is true for α-glucan extracted from the cell wall of *Mycobacterium tuberculosis* (Gagliardi et al., 2007), where this polysaccharide is closely related to the impairment of CD1a lipid antigen presentation, limiting the activation of CD1-restricted lipid-specific T lymphocytes. As a matter of fact, CD1a participation in Pb antigenic lipids presentation to T-cells has not yet been assessed. However, it is possible that the α-glucan present in Pb cell wall may subvert the development of CD1a^+^ population, impairing lipid presentation.

We also observed that both cell wall fractions induced down-regulation of DC-SIGN expression in CD1a^–^ cells (F1) and CD1a^+^ cells (F2). DC-SIGN is a C-type lectin receptor restricted to DCs. Likewise antigen recognition, DC-SIGN participates in DCs migration and T-cell priming (Švajger et al., 2010). Other pathogens make use of DC-SIGN to subvert DCs functions in order to escape immune surveillance, such as HIV-1 and *M. tuberculosis* (van Kooyk et al., 2003; Gagliardi et al., 2007). HLA-DR expression was also inhibited by both F1 and F2, suggesting an impairment of antigen presentation capacity. While CD80 expression was inhibited by both cell wall fractions in CD1a^+^ cells, F2 fraction upregulated its expression in CD1a^–^ cells. Similarly to CD80 upon F2 treatment, CD83 expression was increased in CD1a^–^ cells from both F1 and F2 treatments, although levels remained unaltered in CD1a^+^ cells. Corroborating these data, infection of monocytes with *M. tuberculosis* and Bacillus Calmette-Guérin (BCG), but not with *Mycobacterium avium*, induced lack of CD1a, CD1b and CD1c, presence of maturation markers CD86 and CD83 and down-regulation of CD80 and MHC class II (Gagliardi et al., 2002; Mariotti et al., 2002), and this effect was correlated with α-glucan present in the cell wall (Gagliardi et al., 2007).

In this study, Pb cell wall fractions were able to modulate inflammatory cytokine production by DCs. Both F1 and F2 inhibited production of TNF. In PCM, TNF is required for the persistence of well-formed granulomas and NO production (Figueiredo et al., 1993) and contrarily, it is highly induced in mice after *in vivo* challenge with F1 (Silva et al., 1994). F1 also induced a decrease in IL-12 and an increase in IL-10 and IL-6 by DCs. The same pattern of cytokine production was seen after monocyte differentiation into DCs upon *Candida albicans* β-glucan stimulation (Nisini et al., 2007). Increased IL-10 and low levels of IL-12 downregulate Th1 cell activity, which have a negative outcome for host protection during PCM. F2 stimulation induced decreased IL-8 and increased IL1β levels. These results demonstrate a mixed pattern of cytokine production upon DCs differentiation in the presence of Pb cell wall fractions, with both up- and downregulation of inflammatory mediators.

Our data corroborates previous studies that demonstrated that Pb infection alters DCs maturation, leading to a T cell-mediated response that could influence the susceptibility to this pathogen (Ferreira et al., 2004, 2007; Ferreira and Almeida, 2006; Fernandes et al., 2015). We speculate that, when monocytes migrate to the infection site, they interact with components of the fungal cell wall, especially α-glucans, and undergo an inadequate differentiation into CD1a^–^ DCs. Further studies are required in order to better understand how Pb cell wall α-glucan influences the host immune response during PCM. To the best of our knowledge, this is the first report that demonstrates the influence of Pb cell wall α-glucan in the differentiation and maturation of human DCs.

## Data Availability Statement

The raw data supporting the conclusions of this manuscript will be made available by the authors, without undue reservation, to any qualified researcher.

## Ethics Statement

The studies involving human participants were reviewed and approved by the Blood Center of Salvador, HEMOBA, Brumado, Brazil, authorization number 100/2006. The patients/participants provided their written informed consent to participate in this study.

## Author Contributions

AS performed the research, analyzed and interpreted the data, and wrote the manuscript. CF designed and performed the research and analyzed the data. NS, NT, CS, MJ, PV, and CM performed the research. CB and AB designed the research, analyzed and interpreted the data, and wrote the manuscript.

## Conflict of Interest

The authors declare that the research was conducted in the absence of any commercial or financial relationships that could be construed as a potential conflict of interest.
